# Dosimetry evaluation of SAVI-based HDR brachytherapy for partial breast irradiation

**DOI:** 10.4103/0971-6203.62127

**Published:** 2010

**Authors:** Sivasubramanian R. Manoharan, R. Rodney Rodriguez, Vidya S. Bobba, Mukka Chandrashekar

**Affiliations:** Department of Physics, 21^st^ Century Oncology-Redding Cancer Treatment Centre, Redding, CA-96001, USA; 1 Radiation Oncology, 21^st^ Century Oncology-Redding Cancer Treatment Centre, Redding, CA-96001, USA; 2 Department of Physics, Jawaharlal Nehru Technological University, Hyderabad, AP, India

**Keywords:** Accelerated partial breast irradiation, high dose rate, SAVI

## Abstract

Accelerated partial breast irradiation (APBI) with high dose rate (HDR) brachytherapy offers an excellent compact course of radiation due to its limited number of fractions for early-stage carcinoma of breast. One of the recent devices is SAVI (strut-adjusted volume implant), which has 6, 8 or 10 peripheral source channels with one center channel. Each channel can be differentially loaded. This paper focuses on the treatment planning, dosimetry and quality assurance aspects of HDR brachytherapy implant with GammaMed Plus HDR afterloader unit. The accelerated PBI balloon devices normally inflate above 35 cc range, and hence these balloon type devices cannot be accommodated in small lumpectomy cavity sizes. CT images were obtained and 3-D dosimetric plans were done with Brachyvision planning system. The 3-D treatment planning and dosimetric data were evaluated with planning target volume (PTV)_eval V90, V95, V150, V200 skin dose and minimum distance to skin. With the use of the SAVI 6-1 mini device, we were able to accomplish an excellent coverage — V90, V95, V150 and V200 to 98%, 95%, 37 cc (<50 cc volume) and 16 cc (<20 cc volume), respectively. Maximum skin dose was between 73% and 90%, much below the prescribed dose of 34 Gy. The minimum skin distance achieved was 5 to 11 mm. The volume that received 50% of the prescribed radiation dose was found to be lower with SAVI. The multi-channel SAVI-based implants reduced the maximum skin dose to markedly lower levels as compared to other modalities, simultaneously achieving best dose coverage to target volume. Differential-source dwell-loading allows modulation of the radiation dose distribution in symmetric or asymmetric opening of the catheter shapes and is also advantageous in cavities close to chest wall.

## Introduction

Accelerated partial breast irradiation (APBI) has been performed with multiple methods, which include multi-catheter brachytherapy,[1] Mammosite balloon brachytherapy[2,3] and 3-D conformal external beam therapy.[4] Multi-catheter brachytherapy requires high expertise in the field. Mammosite brachytherapy has been widely accepted and is technically much easier to perform, but the dose distribution is spherically symmetric with single dwell position.[5] Multiple dwell positions in Mammosite balloon add the cumulative total dose, dose shaping to some extent to the patient anatomy, but balloon asymmetry or inadequate balloon-to-skin distance is really a matter of concern. The strut-adjusted volume implant, SAVI (Cianna Medical, Aliso Viejo, California), combines the advantages of both Mammosite and multi-catheter; it uses multiple peripheral struts with central-loading single catheter.[6,7]

The applicator was inserted as a special separate procedure after 4 weeks of lumpectomy for those patients selected for this study. The dose of 34.0 Gy in 10 twice-daily fractions was prescribed to a planning target volume (PTV) created by a three-dimensional 1.0-cm expansion of the cavity. This PTV was then truncated by 5 mm from skin to limit skin dose, and a point dose constraint of 100% of the prescribed dose was assigned to the skin. In this paper, we present the treatment planning criteria and quality assurance (QA) guidelines followed by us to get the best dosimetry.

## Materials and Methods

**Figure 1:**
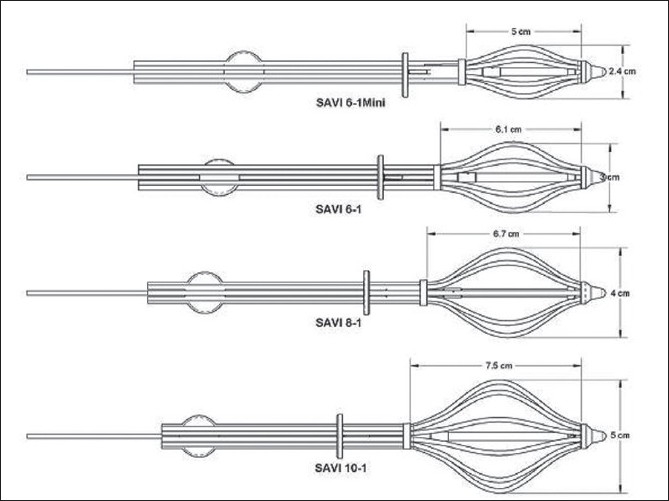
Choice of SAVI sizes; courtesy: Cianna Medical

**Figure 2:**
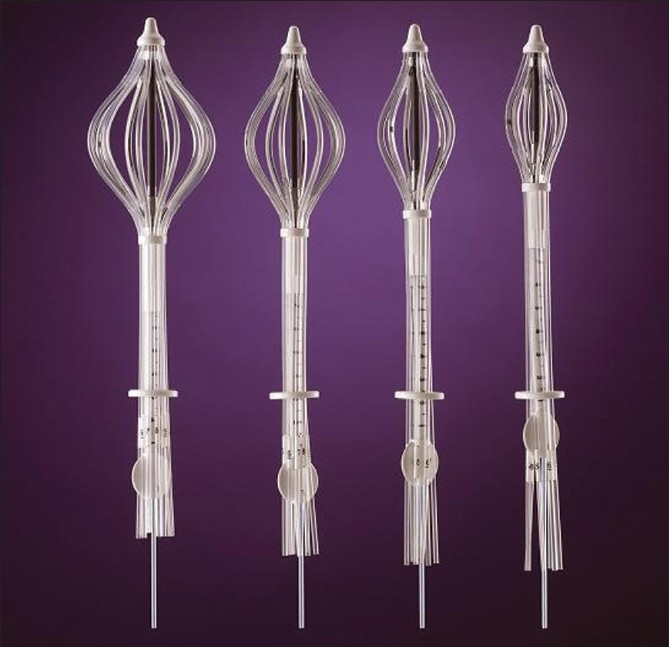
SAVI sizes with peripheral struts expanded; courtesy: Cianna Medical

**Figure 3:**
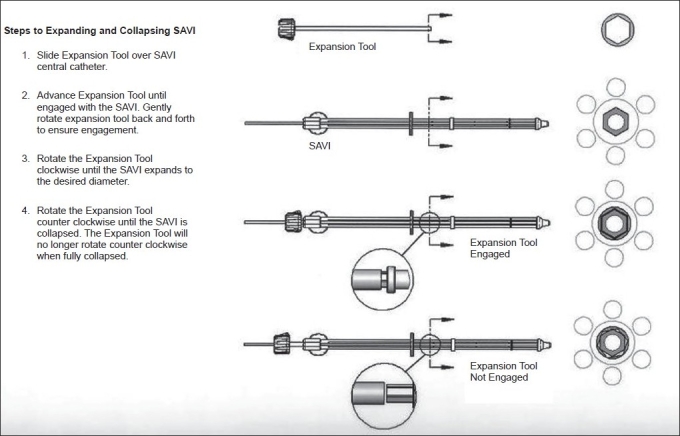
Expansion tool usage [courtesy: Cianna Medical]

The applicator SAVI consists of a central strut surrounded by 6, 8 or 10 peripheral struts, depending on the size of the device. The size specifications for SAVI 6-1 mini applicator are as follows: diameter 2.4 cm with long axis 5.0 cm. SAVI 6-1 applicator has diameter 3.0 cm with long axis 6.1 cm. SAVI 8-1 applicator has diameter 4.0 cm with long axis 6.7 cm. SAVI 10-1 applicator has diameter 5 cm and long axis 7.5 cm [Figures 1 and 2]. Reference chart of SAVI preparatory fill volume in comparison with applicator model is shown in Table 1. The device can be collapsed and expanded to the sizes mentioned above. Usually the device is inserted in the collapsed position; and by clockwise rotation of the knurled knob at the proximal end of the expansion device, as shown in Figure 3, the peripheral struts are expanded. A fixed connecting hub is located at the end of the applicator. An expansion tool can slide over the central strut and can be used to expand the device after the insertion of the device into the lumpectomy cavity. The struts’ expansion into the surrounding tissue provides a pressure fit into the cavity. We observed some tissue invagination in between the struts. The expansion tool was removed after applicator insertion. It was reinserted at each treatment and removed after the treatment delivery without any change to the device expansion. In this way, in case of an emergency the device can be easily collapsed and removed. This allows more flexibility of the device since the applicator will be in place for next 5 days. The struts 2, 4 and 6 have radiopaque markers for identification, which helps during 3-D reconstruction process.

**Table 1:** Selection of SAVI sizes based on diameter, SAVI prep volume and long axis of cavity

*Long axis of cavity in cm*	*Diameter of cavity in cm*		
	*2-3*	*3-4*	*4-5*
2-3	SAVI prep (20 cc) 6-1 Mini		
3-4	SAVI prep (20 cc) 6-1 Mini	SAVI prep (20 cc) 6-1 Mini	
4-5	SAVI prep (20 cc) 6-1 Mini	SAVI prep (20 cc) 6-1 Mini	SAVI prep (30 cc) 6-1
5-6	SAVI prep (30 cc) 6-1	SAVI prep (30 cc) 6-1	SAVI prep (40 cc) 6-1
6-7	SAVI prep (30 cc) 6-1	SAVI prep (40 cc) 8-1	SAVI prep (40 cc) 8-1
7-8	SAVI prep (40 cc) 8-1	SAVI prep (60 cc) 10-1	SAVI prep (60 cc) 10-1

Courtesy: Cianna Medical

### Patient selection

Patients to be treated with APBI using the SAVI device were first chosen according to the Professional Medical Society consensus guidelines, shown in Table 2.

**Table 2:** Patient selection criteria for accelerated partial breast irradiation

	*ABS[11]*	*ASBS[12]*	*ACRO[13]*	*ASTRO[14]*	
				Suitable	Cautionary
Age	≥50	≥45	≥45	≥60	50-59
Diagnosis	Unifocal, invasive ductal carcinoma	Invasive ductal carcinoma or ductal carcinoma *in situ* (DCIS)	Invasive ductal carcinoma or DCIS	Invasive ductal or other favorable subtypes (i.e., mucinous, tubular, colloid)	Pure DCIS ≤3 cm
Tumor size	≤3 cm	≤3 cm	≤3 cm	≤2 cm	2.1-3.0 cm
Surgical margins	Negative microscopic surgical margins of excision	Negative microscopic surgical margins of excision	Negative microscopic surgical margins of excision	Negative by at least 2 mm	Close (<2 mm)
Nodal status	NØ	NØ	NØ	NØ (i-, i+)	

Pre-treatment CT evaluation

After careful evaluation of the patient for HDR brachytherapy, the tumor bed size is evaluated by preoperative CT or ultrasound. We used preoperative CT to determine all aspects of the implant. This allowed us to anticipate what size of applicator was to be used, following the manufacturer’s recommendations of SAVI size in relation to the target volume. The tumor bed size evaluation determined the size of the applicator, with its long axis and angle of entry. According to our experience it can be stated that if the applicator is not parallel to the axial scan, it will be better viewed without any confusion and the reconstruction becomes simple.

Procedure involved during pre-placement CT evaluation

1. CT scan of the breast to be treated, with ≤ 3-mm slices and no gap between slices.
2. Scan field-of-view must include the entire cavity, including 2 to 3 cm superiorly and inferiorly for extra coverage.
3. Patient arms up with breast board.
4. CT data should be transferred to the planning system (in our case, it was to Brachyvision planning system).
5. Evaluation of the cavity by surgeon and oncologist for treatment procedure, including parameters like volume of the cavity, long and short axes measurement.
6. The images are rotated until the long axis is best seen in two orthogonal views, which helps to assess the best point of entry and angle of insertion.
7. Recording these points helps in future assessment.
8. Determine the appropriate size of the SAVI applicator to be used and inform the concerned surgeon.

### Implant

After the preoperative determination, the information obtained was used for the selection of the SAVI device size for procurement and for surgical placement. The SAVI applicator was surgically implanted under ultrasound guidance. A CT scan image was acquired immediately after the implant surgery for treatment planning purposes. Patient positioning needs to be reproducible since every day a CT scan for the device area is required to confirm the size of the applicator and also to rule out any inversion of the struts. Often patient rotation can lead to wrong interpretation of the size of applicator based on the CT image. Thus reproducibility is important for implant verification, and also the patient has to be treated in the same position. Each day of the treatment, the CT images were obtained and compared with the first-day image that was used for initial treatment planning. Usually the surgical clips are placed for reproducibility verification, but the surgical clips sometimes sit in between two lumens, which cannot be visualized. We, as always, determined the applicator reproducibility based on the expanded diameter of the applicator from the initial planning CT, and the distance from the skin surface to the fixed point on the central catheter was measured and was verified for daily pre-treatment. We used the breast board for our patient immobilization, which reduced the setup time and it was quick to reproduce. We took 3-mm slice width scans for our patients with AP and lateral scout views, which were compared with the treatment-day scout image.

**Figure 4:**
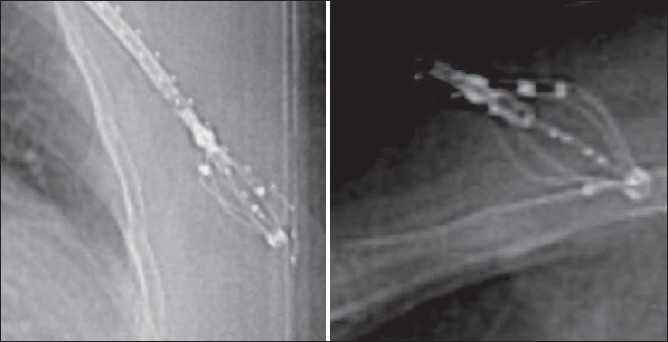
AP and lateral scout images

During initial CT simulation with SAVI implant, the size of the applicator can be adjusted if desired and the patient scanned again. All the catheter protectors in the lumen were removed to insert the marker wires to visualize the distal ends of the lumen for later planning. AP and lateral scout images are as shown in Figure 4. The planning CT data set with 3-mm slice width were obtained with no gap between the slices. The entire ipsilateral breast was scanned with breath-hold technique to reduce artifacts and image blurring. After the CT procedure, reinsertion of all the catheter protectors in the SAVI catheter lumens with the cap is important to avoid any blocks or kinks. AP and lateral scout images were used as reference images for daily pre-treatment evaluation. It is important to verify prior to each treatment fraction that the SAVI implant is in the same position as it was during the treatment planning CT to avoid a rotation of the applicator. Breast board allows reproducibility of the patient position.

### Treatment planning and dosimetry

**Figure 5:**
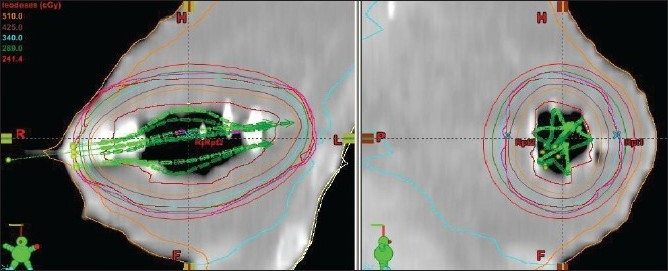
SAVI 6-1 applicators identified with contours and dose distribution

**Figure 6:**
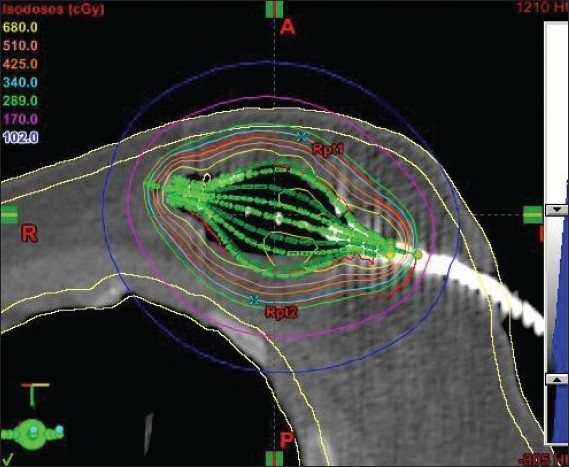
SAVI 8-1 struts identified with dose distribution

The CT images were sent to the Brachyvision planning system, and 3-D reconstruction was done. The cavity contour was drawn by the radiation oncologist. The 1-cm uniform positive expansion of “cavity” creates PTV but the cavity volume is not included. From the PTV contour, PTV_eval contour was drawn by subtracting the skin and chest wall, since the dose could be modulated, and thus PTV_eval conformed to the patient’s anatomy. The PTV_eval is defined as difference between the expanded volume and the cavity volume. The multi-planar reconstruction within the Brachyvision planning system made the catheter reconstruction an easy task. Planning was a little more time consuming than with Mammosite balloon since multiple struts needed to be located correctly and optimized. This PTV_eval was analyzed by the radiation oncologist to ensure the adequacy of dose coverage to the tumor bed region, as shown in Figures 5 and 6.

We performed planning after the implant with initial CT scan; but in case of any change in position of the applicator, re-planning is absolutely necessary to ensure dose coverage to the volume, low dose to skin and normal structures as per the planning criteria. Re-planning is also necessary if there is change in position of the strut(s), change in size of the applicator and applicator position.

### Dose optimization goals

1. PTV_eval: 95% of volume receives ≥ 95% of prescribed dose.
2. PTV_eval: ≥ 200% of dose must be < 20 cc absolute volume.
3. PTV_eval: ≥ 150% of dose must be < 50 cc absolute volume.
4. Skin dose should be < 100% of the prescribed dose.
5. Lung dose should be < 75% of the prescribed dose.

### Quality assurance

**Figure 7:**
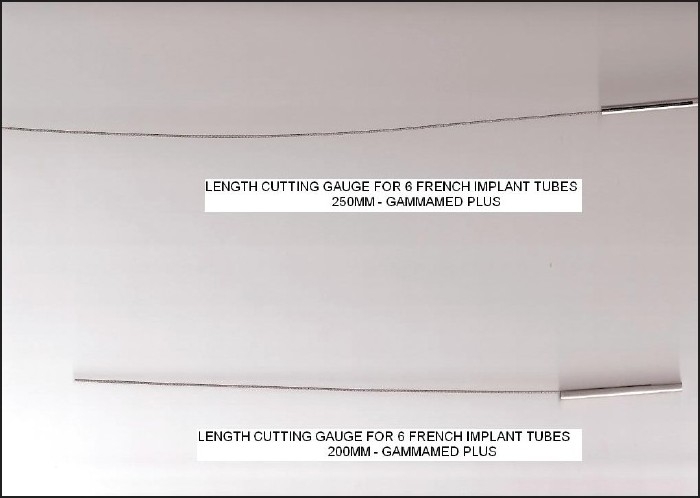
Length-cutting gauge for SAVI applicator

Each applicator connected to the GammaMed Plus HDR afterloader machine has a total length of 1300 mm including the source guide tube length. The center channel of SAVI has a length of 250 mm without the source-connecting guide tube, and the peripheral channels are 200 mm in length. Each of the channels is measured with length-cutting gauge device [Figure 7] to check and correct the length. The total length was also measured before the delivery of each fraction to the patient after connecting the guide tubes to the lumens with the help of the length gauge. Each lumen length along with the source guide tube was measured to confirm that the total length was 1300 mm. Sometimes we found there was discrepancy in the length of the treatment; and in such cases, we adjusted the source guide tube to the correct length or cut the end of the lumen to match the exact length of 1300 mm.

Also care needs to be taken during the planning stage to determine the initial position of the applicator. This was achieved with multi-planar reconstruction of the planning system, along with the guidelines given by the manufacturer. Pre-treatment procedures included the skin-to-hub measurement and the rotational motion or strut integrity check, as discussed early. The expansion tool was inserted over the central lumen, so that if the source tucked in one of the struts, the applicator could be collapsed quickly to remove it from the patient and be kept in the lead emergency storage container. The transfer guide tubes can be numbered since there is every possibility of mistake by interchanging the transfer guide tubes with the SAVI applicator struts, and hence extreme care is necessary. From the safety point of view, it is a good practice to label the transfer guide tubes, and following this label throughout the procedure is essential.

Post-treatment procedures included survey of the patient to assure that the source had been moved out from the patient and was back in the safe parking position of the HDR unit. Also radiation survey of the treatment room is essential. The machine transfer guide tubes were then disconnected from the device, and the expansion tool was removed. If the applicator insertion is parallel to the transverse CT scan images, it would be better to have 1-mm slice thickness to rule out all possible confusion which may lead to wrong reconstruction.

The treatment planning dwell time calculation was independently validated by using an AAPM TG 43[5] one-dimensional point source approximation in an Excel spreadsheet to calculate the dose to the prescription point.

## Results and Discussion

The study considered first 6 patients. All patients were treated with SAVI mini 6-1 size applicator, and the dose delivered was 340 cGy per fraction with 10 fractions being delivered over 5 days. The cavity volumes were less than 30 cc, which prompted us to use SAVI mini 6-1 applicator. The cavity expansion device (CED) was inserted in the central catheter before each treatment, since the CED would be helpful to remove the applicator in case of an emergency, like source struck, etc. The data of dose-volume histogram details of our planned patients are shown in Table 3. The dosimetry data in Table 3 show the treated PTV_eval volume of the patients, which varied between 49 and 91 cc.

**Table 3:** Dose volume histogram obtained for optimized plans

*PTV_eval volume*	*V_90_*	*V_95_*	*V_100_*	*V_150_*	*V_200_*
49-91 cc	97%-98%	93%-95%	90%-92%	24-37 cc	12.8-16.3 cc

(Number of patients = 6).

**Figure 8:**
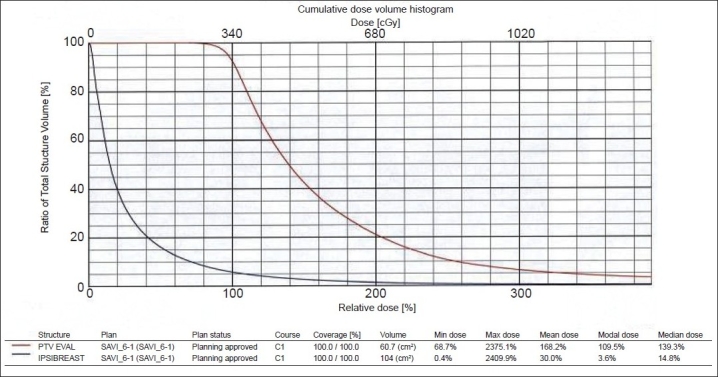
PTV_eval shows 90% dose covers 95% of volume, and 90% volume covers 100% of prescription dose

The PTV_eval volumes for those patients treated with SAVI were within the range 49-91 cc. The PTV_eval V_95_ was 93%-95%, and PTV_eval V_100_ was 90%-92%. (V_95_ is the volume receiving 95% of the prescribed dose or more.) The 100% isodose line conforms to the PTV_eval that lies 5 mm within the skin and pectoral muscle, as shown in Figures 5 and 6. The PTV_eval V_150_ and V_200_ were well below the optimization goal of V_150_ <50 cc and V_200_ <20 cc of the absolute volume. Also the skin dose of the patients was 73%-90% of the prescribed dose, which was not possible in case of Mammosite balloon brachytherapy.[9,10] The Mammosite device has been proven to safely deliver adequate dose to the target tissues in larger breasts with adequate skin sparing, but it does not suit smaller breasts with non-adequate skin margins of less than 5 mm. Patients who are not eligible for Mammosite treatment can be well treated without any compromise on dosimetry. The skin dose levels were determined by using the 3-D volume image data by growing various isodose lines with intersection of the skin. The results of dose-volume histogram evaluation are shown in Figure 8.

## Conclusion

SAVI design with its multiple peripheral catheters allows modulation of the dwell times, which results in a very good conformality to the target while minimizing skin, lung and chest wall irradiation. Following strict quality assurance and dosimetry procedures makes the use of SAVI safe and easily manageable.
